# Assessment of F_v_/F_m_ absorbed by wheat canopies employing in-situ hyperspectral vegetation indexes

**DOI:** 10.1038/s41598-018-27902-3

**Published:** 2018-06-22

**Authors:** Chang-Wei Tan, Dun-Liang Wang, Jian Zhou, Ying Du, Ming Luo, Yong-Jian Zhang, Wen-Shan Guo

**Affiliations:** Jiangsu Key Laboratory of Crop Genetics and Physiology/Co-Innovation Center for Modern Production Technology of Grain Crops/Joint International Research Laboratory of Agriculture and Agri-Product Safety of the Ministry of Education of China, Yangzhou University, Yangzhou, 225009 China

## Abstract

Chlorophyll fluorescence parameter of F_v_/F_m_, as an important index for evaluating crop yields and biomass, is key to guide crop management. However, the shortage of good hyperspectral data can hinder the accurate assessment of wheat F_v_/F_m_. In this research, the relationships between wheat canopy F_v_/F_m_ and *in-situ* hyperspectral vegetation indexes were explored to develop a strategy for accurate F_v_/F_m_ assessment. F_v_/F_m_ had the highest coefficients with normalized pigments chlorophyll ratio index (NPCI) and the medium terrestrial chlorophyll index (MTCI). Both NPCI and MTCI were increased with the increase in F_v_/F_m_. However, NPCI value ceased to increase as F_v_/F_m_ reached 0.61. MTCI had a descending trend when F_v_/F_m_ value was higher than 0.61. A piecewise F_v_/F_m_ assessment model with NPCI and MTCI regression variables was established when F_v_/F_m_ value was ≤0.61 and >0.61, respectively. The model increased the accuracy of assessment by up to 16% as compared with the F_v_/F_m_ assessment model based on a single vegetation index. Our study indicated that it was feasible to apply NPCI and MTCI to assess wheat F_v_/F_m_ and to establish a piecewise F_v_/F_m_ assessment model that can overcome the limitations from vegetation index saturation under high F_v_/F_m_ value.

## Introduction

Photosynthesis was the most important biological process on earth^1^. It was the unique approach by which plants gained energy from the environment. There were three basic effects when light struck a leaf surface: absorption, reflection and transmission. The major part of light was absorbed by the chlorophyll used for photosynthesis, and only a small proportion was de-excited via emission with a longer wavelength as fluorescence, or dissipation as heat^2^. Chlorophyll fluorescence emissions occurred in the red and far-red regions of the plant spectrum (650–800 nm)^3^. Changes in chlorophyll fluorescence parameters of plant leaves could reflect the changes of environmental factors and their effects on plant photosynthetic physiology to a certain extent^4^. In many chlorophyll fluorescence parameters, F_v_/F_m_ was used to characterize the conversion efficiency of the light energy of the PS II reaction center, and its numerical changes were of special significance. However, conventional methods of assessing F_v_/F_m_ from field observations, that involved site-specific complicated parameterizations and calculations, made it difficult to apply over large agricultural areas^5^. These shortcomings could be overcome through the complementary use of hyperspectral measurements of crops, which had several advantages - non-destructive, uniform, could be performed rapidly, and no complicated parameterizations were necessary.

Assessment of F_v_/F_m_ from vegetation indexes (VIs) derived from hyperspectral data, especially remote sensing data, have been reported by several studies^6–10^. For instance, some researchers compared the performance of VIs to assess F_v_/F_m_ of legume crops and reached the conclusion that out of the nine kinds of VIs with a close relationship with F_v_/F_m_, modified soil adjusted vegetation index (MSAVI) performed best^11^. If ground cover was significant, the impact of the background significantly reduced, and F_v_/F_m_ could be better estimated using normalized difference vegetation index (NDVI). Re-normalized difference vegetation index (RDVI) showed an approximate linear relation to F_v_/F_m_ regardless of ground cover. Hyperspectral remote sensing is an important technique to fulfill real-time monitoring for crop growth status based on its superior performance in acquiring vegetation canopy information rapidly and non-destructively. However, the regression analysis was based on only five points making it statistically uncertain. Other scientists used radiative transfer models to estimate F_v_/F_m_ and found that a linear model based on NDVI produced the best estimate results^12^.

Recently, modeled F_v_/F_m_ products based on MODIS have been reported in several studies. Coops *et al*. investigated the increasing availability of time series of F_v_/F_m_ data from MODIS and reported the three dynamic habitat index components varied significantly in their magnitude, principally because of MODIS F_v_/F_m_ estimates being larger than those observed by medium resolution imaging spectrometer (MERIS) F_v_/F_m_^13^. In some previous studies, some researchers compared MODIS F_v_/F_m_ with the measurements for sites in the US and found MODIS Fv/Fm was higher than the ground-measured F_v_/F_m_. Winkel *et al*. compared F_v_/F_m_ from MODIS with *in-situ* measurements for a tropical rainforest in Brazil and concluded that MODIS F_v_/F_m_ was reliable for F_v_/F_m_ assessment^14^. There was a need for an investigation of the performance of VIs in different vegetation ecosystems^15^.

Models based on linear F_v_/F_m_ -NDVI relationships suffered from a major flaw - NDVI saturated at high leaf area index values^16^ and, thus a linear model tended to be insensitive to F_v_/F_m_ changes in such cases^17^. Another issue that needed to be recognized was the scarcity of data for boreal ecosystems. The majority of the above cited studies presented empirical evidence suggesting a functional relationship between F_v_/F_m_ and hyperspectral VIs, and these were mostly focused on forests, grasses (prairies), and some crop types such as rice, wheat and cotton^18^. There were only few reports on quantitatively estimating F_v_/F_m_ for wheat canopies using VIs from remote sensing data^19^. Besides, VIs–F_v_/F_m_ relationships differed from one ecosystem type to another ecosystem due to the influences of vegetation type, strong background signals, canopy structure and spatial heterogeneity^20,21^. Further, existing remote sensing-based F_v_/F_m_ products lacked adequate ground validation, which was critical for establishing the uncertainty and accuracy of such products so that they could be used for guiding crop production practices^22,23^.

This study is motivated by the above-mentioned issues and focuses on exhaustive statistical analyses of F_v_/F_m_ -VIs relationships for wheat canopies, using *in-situ* hyperspectral data collected from a series of field experiments, and aims at determining a practical methodology for estimating F_v_/F_m_ of wheat canopies.

## Results

### Changes in wheat canopy F_v_/F_m_ with growth stage

**Figure 1:**
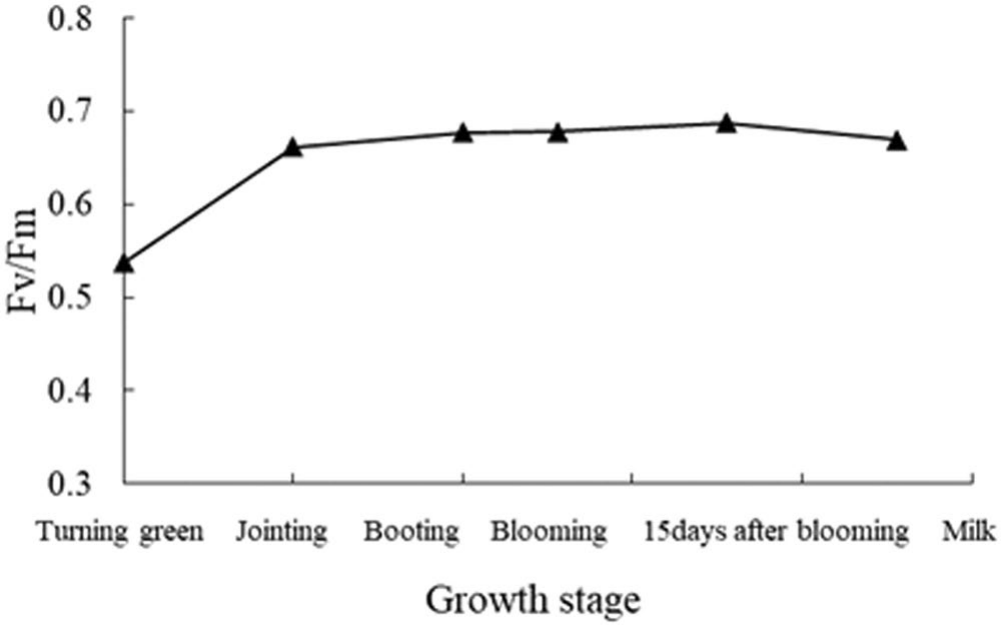
F_v_/F_m_ absorbed by for wheat canopies at different growth stages.

F_v_/F_m_ revealed the progressive increase as the growth of wheat crops at different growth stages (Fig. 1). An initial significant increase in F_v_/F_m_, by about 23.1%, was observed corresponding to crop development from turning stage to jointing stage. However, the further changes in F_v_/F_m_ from booting stage to the milk stage were not significant (2.4%, 0.12%, 1.31% and −2.59%, respectively). Until the blooming stage, F_v_/F_m_ began to increase and reached its maximum value of 0.68. From the blooming stage to the milk stage, F_v_/F_m_ tended to slow, that was saturated.

### VIs-F_v_/F_m_ relationship

Statistically significant correlations between F_v_/F_m_ and VIs were observed in 51 cases out of 56 VIs considered (Table 1), and these were both positive and negative. Positive correlations between VIs and F_v_/F_m_ were generally stronger than the negative ones. F_v_/F_m_ was most strongly correlated to NPCI, MTCI and NDVI [900, 680] -correlation coefficients (*r*) are 0.891, 0.886 and 0.879, respectively. Thus, NPCI, MTCI and NDVI [900, 680] could be identified as three common VIs relatively well correlated to wheat canopy F_v_/F_m_, and these were the post probable VIs of choice for estimating F_v_/F_m_.

**Table 1:** Linear correlation coefficients (*r*) between the F_v_/F_m_ absorbed by wheat canopies and hyperspectral VIs. ^+^ and ^++^ indicate significant difference at 0.05 and 0.01 probability level, respectively.

VIs	*r*	VIs	*r*	VIs	*r*
SR[787, 765]	−0.417^+^	mSRI2	0.814^++^	MSAVI	0.186
SR[415, 710]	0.827^++^	NDI	0.866^++^	OSAVI	0.139
SR[415, 695]	0.675^++^	mNDI	0.707^++^	VARI	0.406^+^
SR[750, 705]	0.686^++^	PSRI	0.813^++^	TCARI	0.654^++^
SR[900, 680]	0.594^++^	RDVI	0.775^++^	WDRVI(a = 0.05)	0.758^++^
SR[801, 670]	0.713^++^	SRPI	0.336^+^	WDRVI(a = 0.1)	0.729^++^
SR[672,550, 708]	0.697^++^	RVI	−0.797^++^	WDRVI(a = 0.2)	0.767^++^
VIopt1	0.815^++^	NPCI	0.891^++^	RGR	0.639^++^
VIopt2	0.839^++^	NPQI	−0.813^++^	NDVI[760, 708]	0.872^++^
PSSR[800, 680]	0.679^++^	SIPI	0.801^++^	NDVI[800, 600]	0.868^++^
PSSR[800, 635]	0.805^++^	MTCI	0.886^++^	NDVI[780, 550]	0.817^++^
PSSR[800, 470]	0.792^++^	MCARI	0.851^++^	NDVI[800, 700]	0.865^++^
ZTM	0.591^++^	GNDVI	0.809^++^	NDIV[900, 680]	0.879^++^
R-M	0.496^++^	MTVI	0.713^++^	TCI/OSAVI	0.052
DI	−0.758^++^	PRI	0.602^++^	MTVI/MSAVI	0.133
DVI	0.724^++^	TVI	0.519^++^	DDI/MSAVI	−0.108
PSND[800, 635]	0.768^++^	TCI	0.485++	MCARI/OSAVI	0.027
PSND[800, 470]	0.765^++^	DDI	0.372^+^	TCARI/OSAVI	0.119
mSRI1	0.737^++^	N*	0.416^++^		

### Establishing the F_v_/F_m_ assessment model based on Vis

A total of 10 VIs were considered for modeling F_v_/F_m_ based on a threshold on VI- F_v_/F_m_ correlation (i.e. *r* > 0.82 in Table 1). These non-linear F_v_/F_m_ assessment models were best represented as exponential functions and were evaluated using their predictive (*R*^2^) and error statistics (RRMSE) (Table 2). Among them, F_v_/F_m_ had the closet exponential relation with NPCI, and had a closer exponential relation with MTCI and NDVI [900, 680], and the models based on NPCI, MTCI and NDVI [900, 680] were capable of estimating the F_v_/F_m_ with *R*^2^ of 0.874, 0.859 and 0.834, respectively, with RRMSE of 0.109, 0.116 and 0.126, respectively, with the assessment accuracy of 89.1%, 88.4% and 87.4%, respectively. Furthermore, according to comparisons of *R*^2^, RRMSE and assessment accuracy, it was more suitable to assess wheat canopy F_v_/F_m_ by NPCI and MTCI than by NDVI [900, 680].

**Table 2:** Quantitative relationships between the F_v_/F_m_ absorbed (*y*) by wheat canopies and hyperspectral VIs (*x*).

VIs	Model	*R* ^2^	RRMSE
SR[415, 710]	y = 0.1037e^3.0714x^	0.719^++^	0.184
VIopt2	y = 0.2164e^0.9382x^	0.734^++^	0.176
MTCI	y = 1.2577e^1.9453x^	0.859^++^	0.116
NDI	y = 0.2361e^2.7634x^	0.773^++^	0.148
NPCI	y = 0.1843e^1.5271x^	0.874^++^	0.109
MCARI	y = 1.2543e^2.1641x^	0.745^++^	0.169
NDVI[760, 708]	y = 0.4925e^2.0015x^	0.809^++^	0.135
NDVI[800, 600]	y = 0.9325e^1.8542x^	0.781^++^	0.142
NDVI[800, 700]	y = 1.7162e^1.1539x^	0.769^++^	0.151
NDIV[900, 680]	y = 2.0192e^1.1934x^	0.834^++^	0.126

^++^ indicates significant difference at the 0.01 probability level.

### Saturation analysis of Vis

**Figure 2:**
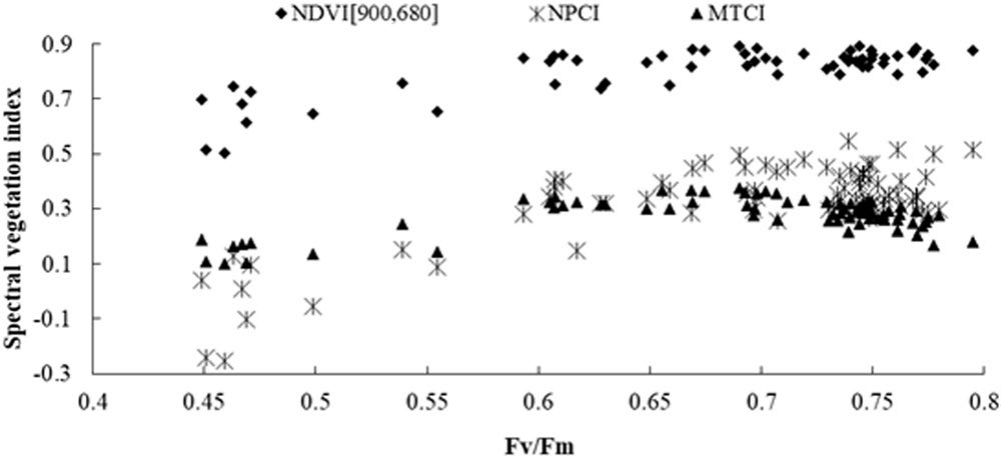
Changes of NPCI, MTCI and NDVI [900, 680] with the F_v_/F_m_ for wheat canopy (*n* = 76).

All three VIs in Fig. 2, i.e. NPCI, MTCI and NDVI [900, 680], which had the strongest relationship to F_v_/F_m_, were increased progressively as F_v_/F_m_ was increased to about 0.61. Beyond this point, NPCI and NDVI [900, 680] values started leveling off at 0.65 and 0.61 respectively, which was also known saturation. On the other hand, MTCI displayed a different tendency when F_v_/F_m_ value was higher than 0.61. Based on this information, a reliable F_v_/F_m_ model could be constructed with NPCI as the regression variable before the saturation point sets in (*F*_*v*_*/F*_*m*_ ≤ 0.61), and with MTCI as while MTCI as the regression variable after the saturation point (*F*_*v*_*/F*_*m*_ > 0.61).

**Figure 3:**
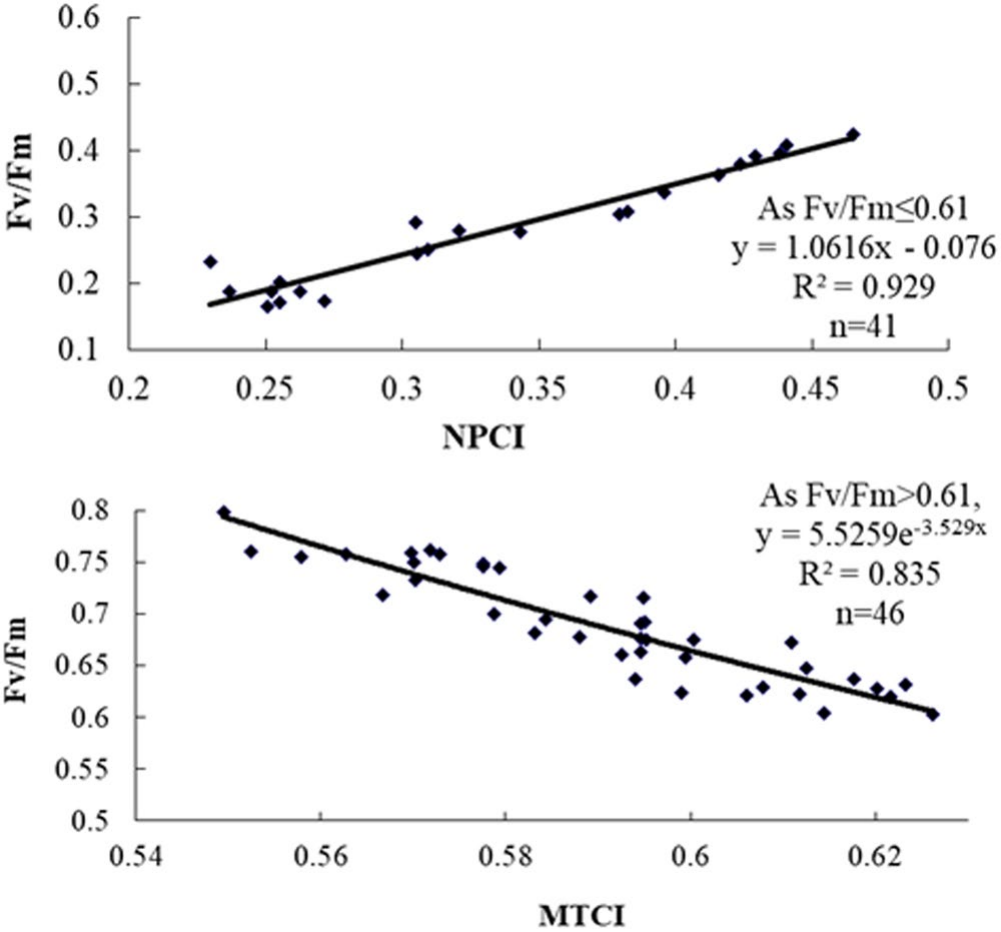
Hyperspectral VIs-based assessment models of F_v_/F_m_ absorbed by wheat canopies. Shown here are the (**A**) NPCI- F_v_/F_m_ model (*F*_*v*_*/F*_*m*_ ≤ 0.61) and (**B**) MTCI- F_v_/F_m_ model (*F*_*v*_*/F*_*m*_ > 0.61). ^++^ indicates significant difference at 0.01 probability level.

Based on the aforementioned research results, the piecewise hyperspectral assessment model of F_v_/F_m_ was built according to the range of F_v_/F_m_ value in Fig. 3. Namely, if F_v_/F_m_ ≤ 0.61, NPCI should be used to assess F_v_/F_m_, and the assessment model was y = 1.0616 × −0.076, *R*^2^ = 0.929 (*p* < 0.01); if F_v_/F_m_ > 0.61, MTCI should be used to assess F_v_/F_m_, and the assessment model was y = 5.5259e^−3.529x^, *R*^2^ = 0.835 (*p* < 0.01).

### Evaluation of VIs-based F_v_/F_m_ model

**Figure 4:**
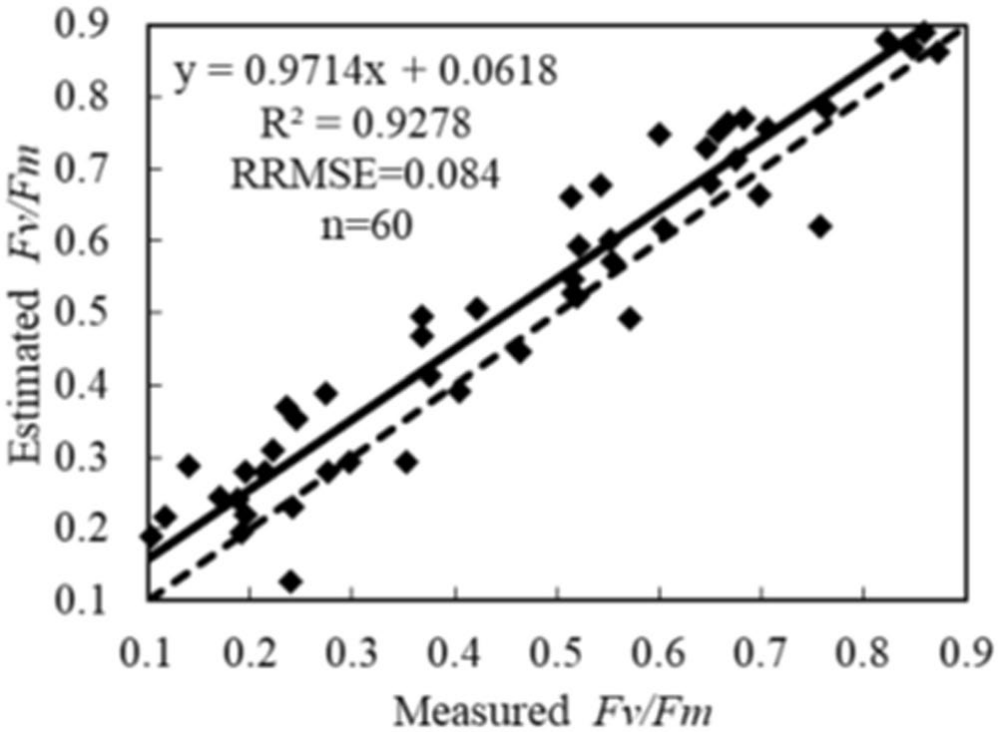
Evaluation of the assessment capability of the piecewise model for the F_v_/F_m_ in wheat canopy. ^++^ indicates significant difference at 0.01 probability level. The solid and dashed lines represent the actual and 1:1 relation between estimated and measured value of F_v_/F_m_, respectively.

A total of 60 samples observed from the experiments in 2017 were used to test the hyperspectral VIs-based assessment model of F_v_/F_m_. The estimated and measured F_v_/F_m_ cross-resistance almost coincided with 1:1 relation line shown in Fig. 4 for comparison. At low F_v_/F_m_ values, estimated value might be underestimated. As F_v_/F_m_ increased, estimated values were closer to the measured values. The *R*^2^, RRMSE and assessment accuracy values of the piecewise F_v_/F_m_ model were 0.9278, 0.084 and 91.6%, respectively. Compared with the assessment models based on only NPCI, MTCI and NDVI [900, 680] in Table 3, the assessment accuracy values of the piecewise F_v_/F_m_ model in different ranges of the F_v_/F_m_ increased by 11.3%, 13.9% and 16.4%, respectively. In conclusion, the piecewise model based on NPCI and MTCI, used to assess F_v_/F_m_, can not only improve the assessment accuracy, but also solve the saturation problems that occurred in NPCI and NDVI [900, 680].

**Table 3:** Definition of hyperspectral VIs evaluated in the study^30^.

VIs	Abbreviation	Algorithm
Simple ratio 1	SR[787, 765]	R_787_/R_765_
Simple ratio 2	SR[415, 710]	R_415_/R_710_
Simple ratio 3	SR[415, 695]	R_415_/R_695_
Simple ratio 4	SR[750, 705]	R_750_/R_705_
Simple ratio 5	SR[900, 680]	R_900_/R_680_
Simple ratio 6	SR[801, 670]	R_801_/R_670_
Simple ratio 7	SR[672, 550, 708]	R_672_/(R_550_ * R_708_)
Optimized vegetation index 1	VIopt1	R_760_/R_730_
Optimized vegetation index 2	VIopt2	100 * (lnR_760_ − lnR_730_)
Pigment specific simple ratio 1	PSSR[800, 680]	R_800_/R_680_
Pigment specific simple ratio 2	PSSR[800, 635]	R_800_/R_635_
Pigment specific simple ratio 3	PSSR[800, 470]	R_800_/R_470_
Zarco-Tejada & Miller	ZTM	R_750_/R_710_
Red-edge model index	R-M	(R_750_/R_720_) − 1
Difference index	DI	R_800_ − R_550_
Difference vegetation index	DVI	R_800_ − R_680_
Pigment specific normalized difference 1	PSND[800, 635]	(R_800_ − R_635_)/(R_800_ + R_635_)
Pigment specific normalized difference 2	PSND[800, 470]	(R_800_ − R_470_)/(R_800_ + R_470_)
Modified simple ratio index 1	mSRI1	(R_750_ − R_445_)/(R_705_ + R_445_)
Modified simple ratio 2	mSRI2	(R_800_/R_670_ − 1)/SQRT(R_800_/R_670_ + 1)
Normalized difference index	NDI	(R_800_ − R_680_)/(R_800_ + R_680_)
Modified normalized difference index	mNDI	(R_750_ − R_705_)/(R_750_ + R_705_ − 2 * R_445_)
Plant senescence reflectance index	PSRI	(R_680_ − R_500_)/R_750_
Re-normalized difference vegetation index	RDVI	(R_800_ − R_670_)/SQRT(R_800_ + R_670_)
Simple ratio pigment index	SRPI	R_430_/R_680_
Ratio vegetation index	RVI	(R_790_:R_810_)/(R_640_:R_660_)
Normalized pigments chlorophyll ratio index	NPCI	(R_680_ − R_430_)/(R_680_ + R_430_)
Normalized phaeophytin ization index	NPQI	(R_415_ − R_435_)/(R_415_ + R_435_)
Structure intensive pigment index	SIPI	(R_800_ − R_445_)/(R_800_ − R_680_)
Medium terrestrial chlorophyll index	MTCI	(R_750_ − R_710_)/(R_710_ − R_680_)
Modified chlorophyll absorption in reflectance index	MCARI	[(R_700_ − R_670_) − 0.2 * (R_700_ − R_550_)] * (R_700_/R_670_)
Green normalized difference vegetation index	GNDVI	(R_800_ − R_550_)/(R_800_ + R_550_)
Modified transformed vegetation index	MTVI	1.2 * [1.2 * (R_800_ − R_550_) − 2.5 * (R_670_ − R_550_)]
Photochemical reflectance index	PRI	(R_531_ − R_570_)/(R_530_ + R_570_)
Transformed vegetation index	TVI	0.5 * [120 * (R_750_ − R_550_) − 200 * (R_670_ − R_550_)]
Temperature condition index	TCI	1.2 * (R_700_ − R_550_) − 1.5 * (R_670_ − R_550_) * SQRT(R_700_/R_670_)
Double difference index	DDI	(R_750_ − R_720_) − (R_700_ − R_670_)
Scaled normalized difference vegetation index	N*	(NDVI − NDVI_0_)/(NDVI_S_ − NDVI_0_)
Modified soil adjusted vegetation index	MSAVI	0.5 * [2 * R_800_ + 1 − SQRT((2 * R_800_ + 1)^2-8 * (R_800_ − R_670_))]
Optimal soil adjusted vegetation index	OSAVI	(1 + 0.16) * (R_800_ − R_670_)/(R_800_ + R_670_ + 0.16)
Transformed chlorophyll absorption in reflectance index	TCARI	3 * [(R_700_ − R_670_) − 0.2 * (R_700_ − R_550_) * (R_700_/R_670_)]
Visible atmospherically resistant index	VARI	(R_555_ − R_680_)/(R_555_ + R_680_ − R_480_)
Wide dynamic range vegetation index	WDRVI	(α*R_nir_ − R_red_)/(α * R_nir_ + R_red_), a = 0.05, 0.1, 0.2
Red green ratio	RGR	(R_612_ + R_660_)/(R_510_ + R_560_)
Normalized difference vegetation index 1	NDVI[760, 708]	(R_760_ − R_708_)/(R_760_ + R_708_)
Normalized difference vegetation index 2	NDVI[800, 600]	(R_800_ − R_600_)/(R_800_ + R_600_)
Normalized difference vegetation index 3	NDVI[780, 550]	(R_780_ − R_550_)/(R_780_ + R_550_)
Normalized difference vegetation index 4	NDVI[800, 700]	(R_800_ − R_700_)/(R_800_ + R_700_)
Normalized difference vegetation index5	NDIV[900, 680]	(R_900_ − R_680_)/(R_900_ + R_680_)
Ratio between TCI and OSAVI	TCI/OSAVI	TCI/OSAVI
Ratio between MTVI and MSAVI	MTVI/MSAVI	MTVI/MSAVI
Ratio between DDI and MSAVI	DDI/MSAVI	DDI/MSAVI
Ratio between MCARI and OSAVI	MCARI/OSAVI	MCARI/OSAVI
Ratio between TCARI and OSAVI	TCARI/OSAVI	TCARI/OSAVI

## Discussion

F_v_/F_m_ was primarily controlled by ground cover and leaf area^24^. Before the jointing stage, F_v_/F_m_ increased significantly (Fig. 1), which was characterized by strong absorption of incoming F_v_/F_m_ as wheat crops grew vigorously, adding leaf area, driven by nitrogen fertilization. This was followed by a lower rate of crop growth (and leaf area expansion), which was captured by the lower rate of F_v_/F_m_ increase. According to agronomic principle of wheat, although the research was lack of F_v_/F_m_ data after the milk stage, it was still available to conclude that leaves started to turn yellow and gradually litter, as the growth period went, and F_v_/F_m_ declined in the combination of wheat’s photosynthetic physiological characteristics. Until full-ripe stage, F_v_/F_m_ was close to 0, because leaves took off green and became withered and died so that they were unable to absorb light energy and the accumulation of dry matter had stopped^25^.

Significant efforts were presently focusing on the use of VIs in general, and NDVI in particular, for estimating vegetation canopy F_v_/F_m_. Furthermore, many studies indicated that VIs were better correlated to F_v_/F_m_ than the reflectance in single wavebands^26,27^, which could be plausibly explained by the fact that VIs could minimize the influence of atmospheric scattering and soil background and enhanced the information of the sensitive wavebands^28^. Similarly, this study found F_v_/F_m_ to be strongly correlated to the majority of VIs (49 out of 56), with NPCI, MTCI and NDVI [900, 680] being the best performing VIs. This result is helpful to provide an important technique for the establishment of perfect wheat photosynthetic groups, the improvement of sunlight energy efficiency and the implementation of cultivation control.

As compared to the previous studies with NDVI, NPCI and MTCI for estimating the F_v_/F_m_ gave the lower RRMSE and higher assessment accuracy than NDVI proposed in several studies. Future research should focus on evaluating the performance of the proposed model over wheat crops grown under a variety of conditions, different wheat varieties, as well as other crop types. This will help in refining the model as a useful tool for informing crop management practices. Efforts should also be made to test this model with data from different sources – field-based spectral measurements, as well as current and future satellite data.

## Conclusion

VIs, like NDVI, were often plagued with saturation at high biomass areas, which was a major disadvantage for VIs-F_v_/F_m_ models. We have addressed this issue by employing the differences in sensitivity of different VIs to F_v_/F_m_ i.e. F_v_/F_m_ had the highest coefficients with NPCI and MTCI. Both NPCI and MTCI were increased with the increase in F_v_/F_m_. However, NPCI value ceased to increase as F_v_/F_m_ reached 0.61. MTCI had a descending trend when F_v_/F_m_ value was higher than 0.61. A piecewise F_v_/F_m_ assessment model with NPCI and MTCI regression variables was established when F_v_/F_m_ value was ≤0.61 and >0.61, respectively. The model increased the accuracy of assessment by up to 16% as compared with the F_v_/F_m_ assessment model based on a single vegetation index. Our study indicated that it was feasible to apply NPCI and MTCI to assess wheat F_v_/F_m_ and to establish a piecewise F_v_/F_m_ assessment model that can overcome the limitations from vegetation index saturation under high F_v_/F_m_ value.

## Materials and Methods

### Experimental design

Four varieties of wheat **-** *Yangmai 13*, *Yangmai 15*, *Yangmai 16* and *Ningmai 9* were used in a field experiment conducted from March to May during the three wheat seasons of 2015, 2016 and 2017 on the Experimental Farm of Yangzhou University, China (119°18′E, 32°26′N). The former crop in the field was rice. The soil is yellow brown soil (Alfisolsin U.S. taxonomy), containing 2.23 g kg^−1^ organic matter, 121.3 mg kg^−1^available nitrogen, 25.9 mg kg^−1^ available phosphorus and 83.7 mg kg^−1^ available potassium in the 0–30 cm soil layer. Canopy spectral parameters were recorded alongside with the quasi-simultaneous measurement of F_v_/F_m_ upon the growing wheat canopies. In order to highlight variations in wheat growth due to biochemical composition changes, three different levels of nitrogen fertilization as urea were implemented, including non-nitrogen fertilization, adequate nitrogen fertilization (450 kg ha^−1^) and heavy nitrogen fertilization (900 kg ha^−1^). There are three replicates for each nitrogen level. The plot size was 4 m × 4 m. Local standard wheat cropping management practices pertaining to water, pest, disease and weed were followed. Training data consisted of 95 and 87 samples in 2015 and 2016, respectively, and test data consisted of 60 samples in 2017.

### Canopy hyperspectral reflectance data

In 2015, six spectral measurements were carried out in the wheat turning green stage (March 7), jointing stage (March 20), booting stage (April 9), blooming stage (April 25), 15 days after blooming stage (May 9), and milking stage (May 18), respectively. All canopy spectrometry determinations were taken at a vertical height of 1.6 m over the canopy under the cloudless or near cloudless condition between 11:00 and 14:00, using an ASD FieldspecPro spectrometer (Analytical Spectral Devices, USA) fitted with 25° field of view fiber optics, operating in the 350–2500 nm spectral region with a sampling interval of 1.4 nm between 350 nm and 1050 nm, and 2 nm between l050 nm and 2500 nm, and with spectral resolution of 3 nm at 700 nm, 10 nm at 1400 nm, selecting the representative, growth-uniform, pest-free plants, placing the probe of sensor down in measuring. A 40 cm × 40 cm BaSO_4_ calibration panel was used for calculation of hyperspectral reflectance. Vegetation and panel radiance measurements were taken by averaging 20 scans at optimized integration time, with a dark current correction at every spectrometry determination.

In 2016, four spectral measurements with 87 test samples were carried out in the wheat turning green stage (March 9), jointing stage (March 22), blooming stage (April 23), and milking stage (May 20), respectively. In 2017, total three spectral measurements with 60 test samples were carried out in the wheat booting stage (April 11), blooming stage (April 22), and 15 days after blooming stage (May 12), respectively. The other practices in 2016 and 2017 were as same as those in 2015.

### Spectral smoothing

Spectral smoothing process was performed in order to remove high frequency noise and the random errors caused by spectral measuring instruments, which enhanced signal to noise ratio. A five-point weighted smoothing method was used to process the raw spectral data^29^. Five-point weighted smoothing method is carried out using Equation (1):

1 $$n=(\frac{{m}_{-2}}{4}+\frac{{m}_{-1}}{2}+\frac{m}{1}+\frac{{m}_{1}}{2}+\frac{{m}_{2}}{4})/25$$

Here, *n* is the weighted average of the intermediate data points in the filter window, namely the smoothed spectrum value, and *m* is the value of unsmoothed data points, namely the original spectral value.

### F_v_/F_m_ measurement

The chlorophyll fluorescence parameters of wheat leaves were determined by modulated fluorescence OS1-FL (Opti-Sciences, Tyngsboro, MA, USA) after the completion of each spectrum. First, the dark adaptation clamp was used to adapt the blade to 10 min, and then the initial light energy conversion efficiency of photosystem II (PS II) F_v_/F_m_ was measured, and the calculation was repeated 9 times each time. The formula is as follows:

2 $${{\rm{F}}}_{{\rm{v}}}/{{\rm{F}}}_{{\rm{m}}}=({{\rm{F}}}_{{\rm{m}}}-{{\rm{F}}}_{{\rm{o}}})/{{\rm{F}}}_{{\rm{m}}}$$

Here, the F_o_ is the basic fluorescence value under the dark adaptation condition; the F_m_ is the maximum fluorescence value under the dark adaptation condition; the F_v_ is the fluorescence value under the variable condition.

### Hyperspectral VIs

In reference to previous studies, based on spectral characteristics of wheat and combined with the physical meaning of spectral index, a total of 56 VIs were considered (Table 3)^30^ related to F_v_/F_m_, leaf area index and chlorophyll (known as an important influence on F_v_/F_m_ absorbed by green vegetation) as the independent variables for establishment of remote sensing assessment models of wheat canopy F_v_/F_m_. Data from the field experiment in 2015 (95 samples) and 2016 (87 samples) were used to develop the regression models, and data from the field experiments in 2017 (60 samples) were used to evaluate the models.

### Statistical analysis

VIs-F_v_/F_m_ relationships were analyzed using a variety of regression models - linear, exponential, logarithmic, and quadratic. Models were ranked based on statistically significant (*p* < *0.05 or 0.01*) correlation coefficients (*r* in case of linear models) and coefficients of determination (*R*^2^ in case of non-linear models). Finally, by plotting the relation figure under the scale 1:1 between estimated and measured F_v_/F_m_ values, the performance of the model was evaluated through the coefficient of determination (*R*^2^) and relative root mean squared error (RRMSE) for the assessment of *in-situ* measured F_v_/F_m_. The higher the *R*^2^ and the lower the RRMSE, the higher the accuracy of the model to assess the F_v_/F_m_. The RRMSE and assessment accuracy are calculated using Equations (3) and (4), respectively:

3 $$RRMSE=\sqrt{\frac{1}{n}\sum _{i=1}^{n}{({y}_{i}-{\hat{y}}_{i})}^{2}}/\frac{1}{n}\sum _{i=1}^{n}{y}_{i}$$

4 $${\rm{Assessment}}\,{\rm{accuracy}}=1-{\rm{RRMSE}}$$

Here, *y*_*i*_ and ${\hat{y}}_{i}$ are the measured values and predicted values of wheat canopy F_v_/F_m_, respectively. *n* is the number of samples.
